# Affordability of Medication Therapy in Diabetic Patients: A Scenario-Based Assessment in Iran’s Health System Context

**DOI:** 10.34172/ijhpm.2020.152

**Published:** 2020-08-22

**Authors:** Leila Zarei, Payam Peymani, Najmeh Moradi, Mehrnaz Kheirandish, Mahtabalsadat Mirjalili, Marziyeh Zare

**Affiliations:** ^1^Health Policy Research Center, Institute of Health, Shiraz University of Medical Sciences, Shiraz, Iran.; ^2^Health Management and Economics Research Center, Iran University of Medical Sciences, Tehran, Iran.; ^3^Assessment and Control of Prescribing and Use of Health Products, Food and Drug Administration, Tehran, Iran.; ^4^Shiraz University of Medical Sciences, Shiraz, Iran.

**Keywords:** Affordability, Diabetes Medicines, Scenario-Based Assessment, Iran

## Abstract

**Background:** Diabetes imposes an enormous burden on patients, families, societies, and healthcare systems. Determining the affordability of medications is an important complicated and vague task, especially in low- and middle-income countries (LMICs). This study aimed to assess the affordability of diabetes medication therapy in Iran’s health system.

**Methods: **This paper presents a scenario-based assessment of the affordability of all registered anti-diabetes medications in Iran in 2017. To this end, 4 medication therapy scenarios were defined as mono, dual, triple, and insulin therapy in accordance with the existing guidelines and clinicians’ opinions. Then the affordability ratio of each treatment scenario was determined for type 1 and type 2 diabetes drawing on the World Health Organization (WHO)/Health Action International (HAI) Methodology. If the affordability ratio for treatment schedules was more than 1, the patients’ out-of-pocket (OOP) expenses exceeded the lowest-paid unskilled government worker (LPGW)’ wage per day, and the treatment was labelled as non-affordable.

**Results: **The results revealed that the mono, dual, and triple (non-insulin) medication therapies in type 2 diabetes were affordable, despite an increase in the dosage or a switch from the monotherapy to the combination therapy of oral medications. However, some treatment scenarios in the triple therapy, including oral plus insulin and some insulin only therapies, were proved to be non-affordable. In type 1 diabetes, only insulin glulisine, detemir, and lispro were non-affordable in monotherapy. Regarding the combination therapy, only isophane insulin with aspart or regular insulin were affordable treatments.

**Conclusion: **Although oral medication therapies were documented to be affordable, insulin therapy, with current coverage conditions, for patients with lowest paid wages and those receiving even less is unaffordable and a major barrier to treatment; hence, policy-maker should consider targeting and more financial protection policies to improve the affordability of insulin therapies among this group of patients.

## Background

**Key Messages**

Implications for policy makers

- For diabetic patients with the lowest paid wages, health policy-maker should consider targeting and more financial protection policies to improve the access to unaffordable long-time/life-time treatments.
- Alongside adopting reimbursement policies to improve affordability of insulins, considering the rising costs of recent antidiabetic agents, further attention should be paid to pricing non-affordable insulins and, also, promoting rational prescription and consumption.
- The results show that the health policy-makers should adopt robust measures to timely identify and subsidize poorer households, as stipulated in the national health insurance policy, to support such patients financially.

Implications for the public

Medication affordability takes on added significance in the case of diabetes, as a chronic non-communicable disease (NCD), because the medicines for their treatment should often be taken for a long time and even lifetime in some cases. To ensure better protection against uncertain financial consequences resulting from any impending catastrophic illness, identifying more catastrophe treatments and targeting patients improve access and affordability in an efficient manner.

According to the World Health Organization (WHO) Guidelines on Country Pharmaceutical Pricing Policies, healthcare decision-makers should find appropriate solutions to manage medicine prices, their availability, and affordability in low- and middle-income countries (LMICs).^1^ Undoubtedly, medication affordability has always been considered as a formidable challenge in healthcare decisions and debates.^2^

 On the other hand, medication is the largest household expenditure item, following food expenditures, in developing countries.^3^ Twenty to 60% of health expenditure in LMICs accounts for medications, which is a significant proportion,^3^ and 90% of individuals living in developing countries have to buy their medicines out-of-pocket (OOP).^4^ It has been reported that more than one-third of the global population cannot afford their necessary medicines^4^; Therefore, policy-makers in LMICs have to address questions about the OOP payments for healthcare services and medications.^2^ Medication affordability takes on added significance in the case of non-communicable diseases (NCDs) as the medicines for their treatment should often be taken for a long time and even lifetime in some cases.^5^

 Diabetes is one of the 4 major NCDs detected by the WHO and accounts for 4% of deaths caused by NCDs and 3% of all global deaths.^6^ The epidemic of diabetes and impaired glucose tolerance in adults is spread worldwide, and its global prevalence has been increasing for the past few decades.^7^ The latest available data estimates that the prevalence of this disease is 8.4% among adults aged 18 to 99 years old, and this value will rise by 9.9% in 2045.^8^ According to the International Diabetes Federation’s report released in 2017, 4 985 000 persons are struggling with diabetes in Iran.^9^ On the other hand, the approximately abrupt rise in healthcare costs associated with diabetes is a formidable challenge to be dealt with the health system. In fact, the average healthcare cost for diabetics is 2.3 times higher than that for non-diabetic patients primarily due to direct healthcare expenditures, loss of productivity as a result of disability, and premature mortality.^10^ Over the last 3 decades, plenty of research has been conducted on the economic burden of diabetes.^11^ A recent systematic review on diabetes treatment cost in LMIC indicates this disease as a high-cost care even though complication types and care for complications varies widely across countries.^12,13^

 Regarding the high-cost care services, some remarkable reasons should be taken into account. However, dramatic changes occurred in the formulation of insulins, and different non-insulin anti-hyperglycemic agents developed in the past century, which affected the diabetes treatment cost.^14^ Now, 11 classes of anti-diabetic medications, approved by the Food and Drug Administration (FDA), are available in the global market for diabetes management.^15^ Another reason is that the multi-morbidity observed in a majority of diabetic patients is associated with an increase in primary healthcare costs.^16^ Undoubtedly, the healthcare systems’ expenditures on diabetes medications has increased worldwide in recent years.^17^For example, the data from the United States shows that higher expenditure on diabetes medications in the past 20 years made an increase in diabetes expenditures.^18^ Furthermore, in European countries, including France, Germany, Italy, Spain, the United Kingdom, the expenditures on insulin and oral antidiabetic medications accounted for 6.2% and 10.5% of total direct cost of diabetes care in 2010, respectively.^19^ Iran has also witnessed an upward trend in the consumption of diabetes medications.^20,21^ A study showed that the consumption of diabetes medications increased from 4.47 in 2000 to 33.54 defined daily doses per 1000 inhabitants per day in 2012.^22^ Similarly, diabetes is the 9th and 16th leading cause of death among Iranian women and men, respectively,^23^ and this inevitably imposes high healthcare costs on the country’s health system.^24^ This issue partly explains why health expenditures have always been one of the major issues discussed in the healthcare policies.^25^ The overall expenditure index raised about 30 times during the past 2 decades in Iran while the growth rate of health expenditures index was 71 times in the health sector. In this regard, total medical expenditures are rapidly approaching 10% of the gross domestic product, crowding out other priorities of Iran’s healthcare system.^26^ Whilst the primary care is financed by Iran’s government, different insurance schemes financially provide secondary/tertiary care services.^27^ While the expenditure on diabetes treatment by healthcare systems has increased in recent years, the affordability of medications for diabetic patients is now a great challenge^17^ since the direct OOP in Iran is higher than that of a majority of other countries across the world though Iran’s healthcare system is insurance-based.^28,29^.Previous studies conducted in Iran have mainly focused on diabetes expenditures.^13,30^ Although the affordability of medications in LMICs, where medicines are often highly-priced with regard to income levels,^31^ has attracted ever-increasing attention, few studies have examined this topic in LIMCs, including Iran. Hence, little data is available on the affordability of diabetes treatment, particularly medication therapy, for patients in LIMCs.^13,32^ Accordingly, given the fact that a significant portion of health expenditures goes to pharmaceutical expenditures and regarding the emphasis of Iran’s National Drug Policy on improving the affordability and accessibility of medications, this study aimed to assess the affordability of antidiabetic medications in Iran comprehensively to estimate the pharmaceutical expenditures incurred by diabetic patients on their medication therapy – to inform policy-makers and provide them with guidelines to develop new policies or improve financial protection policies. Therefore, the specific objectives of the present study were to examine OOP expenditures of diabetic patients under different medication treatment schedules to assess how much they are affordable and what aspects of medication therapy need further attention to promote the accessibility and affordability of treatments in long-term.

## Methods

###  Study Design

 This research was a cross-sectional study assessing the affordability of all registered medications to treat type 1 and 2 diabetes in Iran’s healthcare system in 2017. For this purpose, different individualized medication therapy scenarios were designed based on international clinical guidelines^33,34^ and clinicians’ opinions and then assessed according to the standard treatment approach proposed by WHO and the Health Action International (HAI).

###  Affordability Measurement 

 The WHO/HAI’s methodology in Medicine Prices, Availability and Affordability project was used to measure affordability,^35^ according to which affordability was measured as the required number of days for the wages of the lowest-paid unskilled government worker (LPGW) to be paid for treatment courses. Medications, whose cost exceeded the wage of LPGW per day, were labelled as non-affordable (WHO/HAI methodology). The treatment course was considered as the full course of therapy in the case of acute conditions while the affordability was assessed based on the treatment cost for one month for chronic life-long conditions.

###  Scenario Development 

 To manage diabetes and adapt patients with the therapeutic strategies (eg, oral or injectable medications), the treatment balance between optimal disease management and the other important considerations, including diabetes complications, comorbidities, and patient preferences, is crucial. Accordingly, different treatment scenarios were developed for type 1 and type 2 diabetes to assess the affordability of diabetes medication therapy as follows:

####  Type 2 Diabetes Treatment Approaches

 The management of the type 2 diabetes is associated with many challenges and complications. Individualized approaches to the type 2 diabetes management have been extensively recommended in the most international clinical guidelines as such an optimal hemoglobin A1C should be considered with regard to each patient’s condition.

 International guidelines recommend monotherapy as the first line of medication therapy, except in the case of contraindications or patient intolerance. If treatment with monotherapy does not result in optimal blood glucose levels, then the dual therapy should be initiated. If the dual therapy fails to control the blood glucose, the treatment process are pursued with adding a third agent.^36^ Depending on the type of medication the patient is taking, the triple therapy could be “triple oral therapy” such as metformin, glyburide, pioglitazone, or “triple therapy with insulin,” including bedtime glargine, metformin, and sitagliptin.^37^

 A second and third oral antidiabetic agent such as sulfonylureas, dipeptidyl peptidase 4 inhibitors, and GLP-1 receptor agonists is added if the mono or dual therapy do not result in appropriate blood glucose levels, respectively. Insulin is added or switched where oral therapeutic options plus lifestyle intervention fail. In this regard, the options are isophane insulin (NPH) and short/long-acting insulin analogs such as insulin glargine and aspart.^38^

####  Type 1 Diabetes Treatment Approaches

 Insulin therapy is a fundamental therapy for the type 1 diabetes. Most patients should be treated with multiple daily injections of prandial and basal insulin.^34^

###  Data Collection and Analysis 

 First, a structured form was developed to collect needed data to estimate the cost of medication therapy scenarios. It contained medicine information including generic name, ATC code, dosage forms, dose, defined daily dose, market availability, price, insurance coverage, consumption duration (chronic or acute condition), consumption interval, and the minimum daily wage of an unskilled worker. The data were extracted from formal websites such as WHO, Iran FDA, Iranian Health Insurance Organization, and Ministry of cooperatives labor and social welfare (see Supplementary file 1).

 Then the treatment cost and patients’ OOP were determined for treatment schedules under mono–and combination medication therapy scenarios. The OOP was patient’s payment after deducting health insurance coverage. Generally, the coverage range of medicines is 70%, 90%, and 95% for some type of Insulins.

 Finally, the affordability ratio – patients’ OOP divided by minimum daily wage – was calculated for each treatment schedule. If the ratio was >1, the patient’s OOP for medication therapy was more than one day of LPGW, and the treatment was labelled as non-affordable, otherwise, it was affordable. The minimum daily wage of LPGW in Iran in 2017 was 370 000 Iranian Rials per day, ie, US$8.8, based on the exchange rate of Iran’s Central Bank.

## Results

###  General View 

Table 1 shows the list of antidiabetic medicines in Iran’s healthcare system. Totally, 40 antidiabetic medicines were registered in Iran Drug List by 2017, among which 3 medicines (namely Metformin 750 mg extended-release, linagliptin 5 mg, and exenatide 2 mg extended-release for injection) were excluded from our assessment since the sales data of these medications were not available for 2 previous years leading up to the year of the study, ie, 2017.

**Table 1:** Diabetes medications in IDL in 2017

**Medicine Group**	**Medicine Name**	**Medicine Price** ^a^	**ATC Code**	**Dosage Form**
Alpha glucosidase inhibitor	Acarbose	2500	A10BF01	2
Biguanide	Metformin	900	A10BA02	5
Biguanide + sulfonylurea/thiazolidinedione/dipeptidyl peptidase-4 inhibitor	Metformin + glibenclamide	1200	A10BD02	5
	Metformin + pioglitazone	5000	A10BD05	
	Metformin + sitagliptin	12 300	A10BD07	
Dipeptidyl peptidase-4 inhibitor	Linagliptin	-	A10BH05	1
Dipeptidyl peptidase-4 inhibitor	Sitagliptin	10 000	A10BH01	3
Glucagon-like peptide-1 receptor agonist	Exenatide	-	A10BX04	2
	Liraglutide	1 850 000	A10BX07	
Insulin	Insulin (regular)	140 000	A10AB01	11
	Insulin aspart	295 000	A10AB05	
	Insulin biphasic isophane	72 000	A10AB06	
	Insulin detemir	355 000	A10AB30	
	Insulin glulisin	235 000	A10AE01	
	Insulin isophane	72 000	A10AE04	
	Insulin zinc	-	A10AE05	
Meglitinides	Repaglinide	650	A10BX02	3
Sulfonylureas	Chlorpropamide	65	A10BB01	5
	Glibenclamide	370	A10BB02	
	Gliclazide	1100	A10BB09	
Thiazolidinedione	Pioglitazone	3500	A10BG03	3

Abbreviations: IDL, Iran Drug List; ATC, Anatomical Therapeutic Chemical. ^a^ The price is the least price of generic medicine based on the local currency (Iranian Rial). The health insurance organization usually uses this price as the reference for reimbursement.

###  Results of Scenario-Based Affordability Assessment

####  Type 2 Diabetes

**Figure 1:**
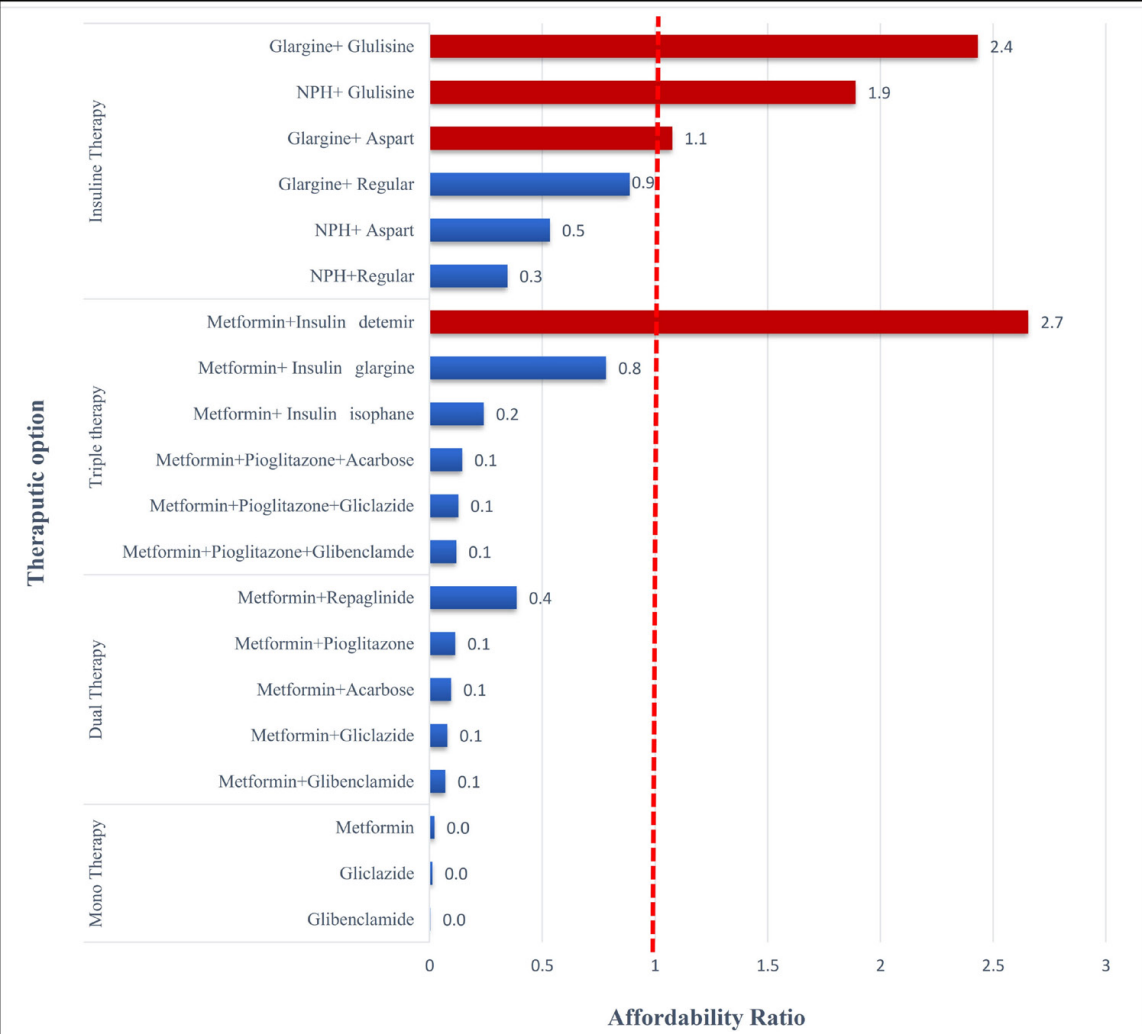
Affordability Ratio in Initial Treatment Scenarios in 4 Therapeutic Options. Abbreviation: NPH, isophane insulin.

 Regarding the recommendation made in clinical guidelines on defining individualized treatment, different dose/schedule adjusted treatment scenarios were developed under therapeutic options for acute and chronic lifelong consumption during a 30-day period: mono, dual, and triple medicine therapy and insulin therapy. Figure 1 shows the patients’ payment for each treatment schedule in term of the wages of LPGW per day. The red dotted line equals one minimum daily wage. If OOP exceeds it, considered non-affordable. In general, there are 4 non-affordable treatment schedules since the patients’ OOP exceeds the one-day minimum wage.

**Figure 2:**
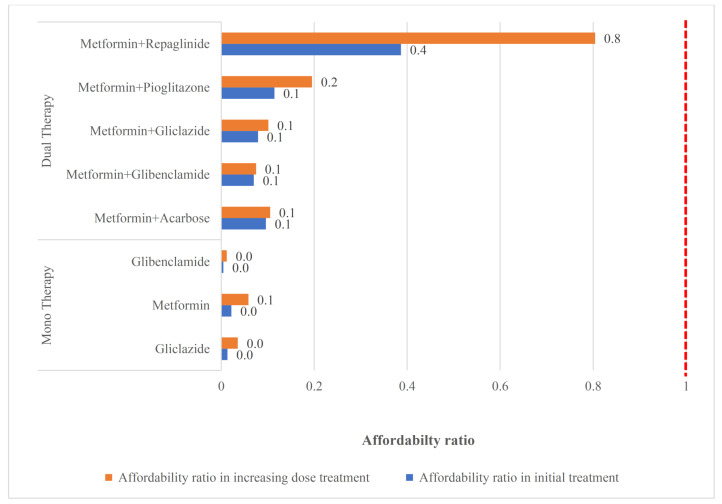
Change in Affordability in Mono and Dual Therapies Due to Failing Initial Therapy.

**Figure 3:**
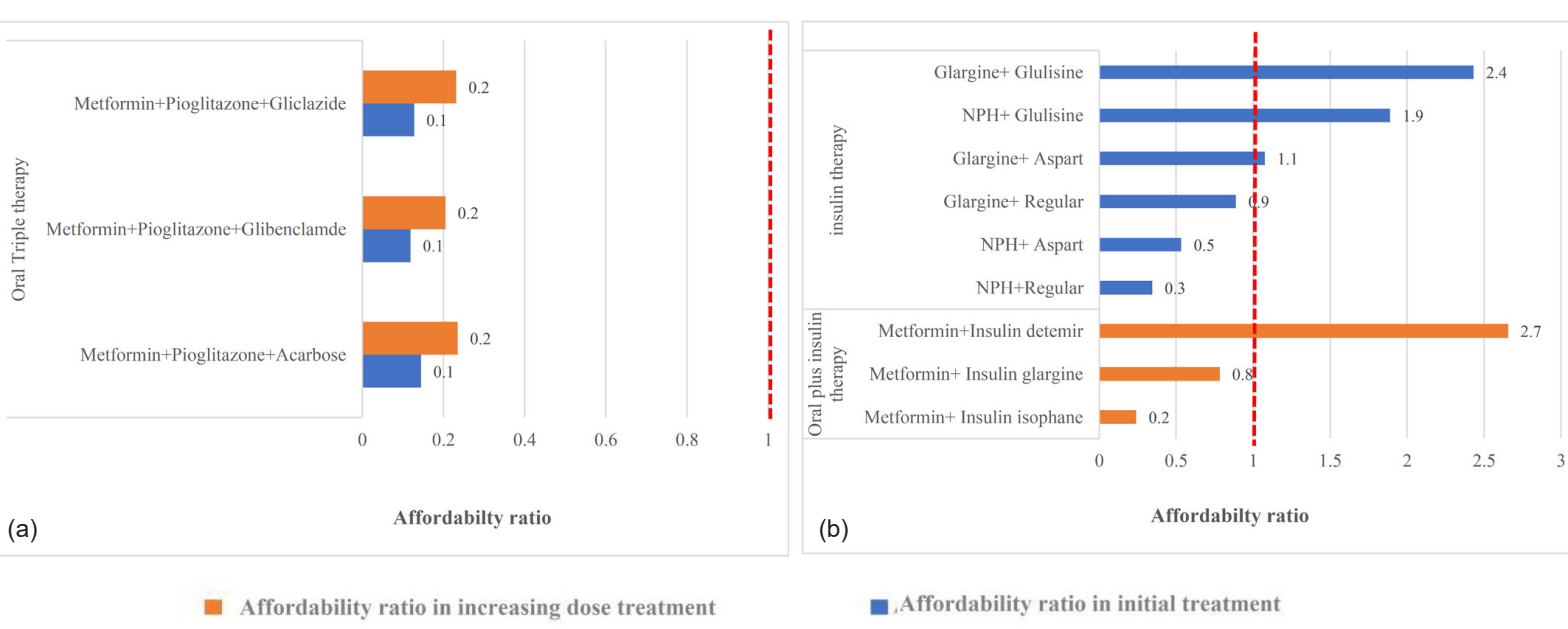
(a) Change in Affordability in Triple Therapy (Insulin Excluded) Due to Failing Initial Therapy. (b) Change in Affordability in Triple Therapy (Insulin Included) Due to Failing Initial Therapy. Abbreviation: NPH, isophane insulin.

**Figure 4:**
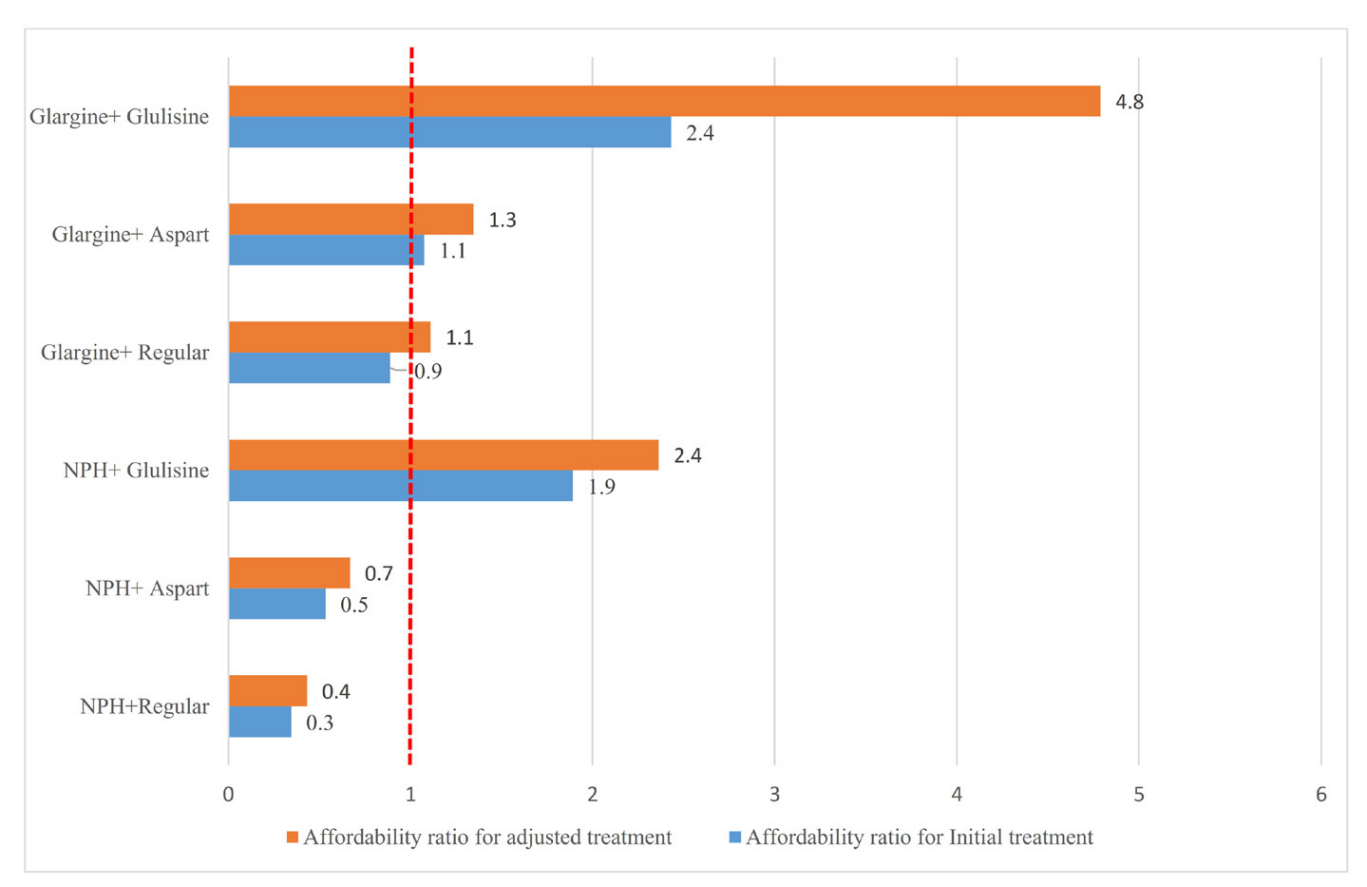
Change in Affordability in Insulin Therapy Due to Failing Initial Therapy. Abbreviation: NPH, isophane insulin.

 In the case of initial treatment failure, the clinician increased doses by 50% after every 7 days in monotherapy and had other dose-adjusted consideration for the combination therapy. Then the next treatment option would be tested if the patient did not respond to the new dosage. Figures 2-4 shows the change of the affordability ratio caused by the failure of treatments (for further details, see Supplementary file 2).

 In the mono and dual therapies, as shown in Figure 2, all options have affordability ratio <1; hence, they are affordable even after an increase in the dosage caused by the failure of the initial treatment. This is because these medicines are usually low-priced medications and enjoy a 70% insurance coverage.

 In the triple therapy, as demonstrated in Figure 3a, all the oral medication therapies are affordable even after an increase in the dosage. Moreover, in combination with the oral plus insulin, all the treatments, with the exception of metformin and detemir, are affordable. With the failure of the treatment and a switch from medicine plus insulin to insulin alone, 50% of the scenarios were non-affordable (Figure 3b). Considering detemir, which was initially non-affordable, one reason could be differences in insurance coverage as it is under insurance coverage by 70%; however, the coverage of glargine and isophane is 90%, thereby making patients pay more.

 The higher percentages of insurance coverage for some insulins are thanks to a memorandum of understanding signed between the health insurance organization and Iran FDA.

 Finally, in the insulin therapy scenarios, 4 out of the 6 treatment scenarios were non- affordable when the dosage increased. Figure 4 shows the affordability ratio in the initial treatment and dose/schedule-adjusted treatment scenarios caused by the failure of initial insulin therapies.

 The results from the scenario-based method showed the affordability of monotherapy with metformin, gliclazide, and glibenclamide, dual therapy with metformin/acarbose, metformin/repaglinide, metformin/glibenclamide, metformin/gliclazide, and metformin/pioglitazone, and triple therapy with metformin/pioglitazone/glibenclamide, and metformin/pioglitazone/gliclazide, and metformin/pioglitazone/acarbose. When the patients did not respond to the triple therapy, metformin and basal insulin, including detemir, glargine, and isophane were administered, or metformin was substituted by a long-acting and a short-acting insulin, including regular, aspart, and glulisine. In the first scenario, all the combinations, except for metformin and detemir insulin, were affordable while the combinations of NPH/glulisine, glargine/aspart, and glargine/glulisine were non-affordable in the second scenario. As for the patients receiving the insulin therapy, the treatment was affordable only in the case of NPH in combination with aspart or regular.

####  Type 1 Diabetes

 Patients with type 1 diabetes were prescribed one basal insulin, including NPH and glargine plus fast-acting insulin such as regular insulin, aspart, or glulisine, at a daily dose of 0.5 unit/kg. If the patient responded to the treatment, the medication therapy continued with the same doses for the rest of the patient’s lifetime. The treatment in this scenario would be only available if the person was taking NPH insulin with regular insulin or aspart Insulin because of its low insurance coverage. As shown in Table 2, according to the guidelines and experts’ opinions, dose/schedule-adjusted scenarios for type 1 diabetes were considered in different therapeutic options during a 30-day period.

**Table 2:** Scenario-Based Affordability Assessment for Type 1 Diabetes

**Medication Therapy**	**Generic Name**	**Dose**	**Dosage Form**	**Dose of Administration/Day**	**Insurance Coverage (%)**	**Affordability Ratio**
Monotherapy	Insulin regular human	100 IU/mL	Vial	40 IU	95	0.23
	Insulin regular human	100 IU/mL (3 mL)	Cartridge	40 IU	90	0.08
	Insulin aspart	100 IU/mL (3 mL)	Pen	40 IU	90	0.32
	Insulin aspart rapid	100 IU/mL (3 mL)	Pen	40 IU	90	0.32
	Insulin biphasic isophane 70+30	100 IU/mL (10 mL)	Vial	40 IU	95	0.02
	Insulin biphasic isophane	100 IU/mL (3 mL)	Cartridge	40 IU	90	0.08
	Insulin isophane	100 IU/mL (5 mL)	Vial	40 IU	70	0.27
	Insulin isophane (NPH) beef	100 IU/mL	Vial	40 IU	70	1.16
	Insulin isophane (NPH) human	1000 IU/10 mL	Vial	40 IU	95	0.23
	Insulin Isophane (NPH) Human	100 IU/mL (3 mL)	Cartridge	40 IU	90	0.08
	Insulin Glargine	300 IU/3 mL	Cartridge	40 IU	90	0.83
	Insulin Glargine	300 IU/3 mL	Pen	40 IU	90	0.96
	Insulin Glulisine	300 IU/3 mL	Vial	40 IU	70	2.29
	Insulin Lispro	100 IU/mL	Pen	40 IU	0	70.03
	Insulin Detemir	100 IU/mL	Injection	40 IU	70	3.45
Combination therapy (50% basal+ 50% rapid or short acting)	NPH + Regular	100 IU/mL	Isophane (Vial)	0.5 IU/kg/d	90	0.43
		100 IU/mL	Regular (Vial)	0.5 IU/kg/d	95	
	NPH + Aspart	100 IU/mL	Isophane (Vial)	0.5 IU/kg/d	90	0.67
		100 IU/mL	Aspart (Pen)	0.5 IU/kg/d	95	
	NPH + Glulisine	100 IU/mL	Isophane (Vial)	0.5 IU/kg/d	90	2.36
		100 IU/mL	Glulisine (Pen)	0.5 IU/kg/d	70	
	Glargine + Regular	100 IU/mL	Glargine (Pen)	0.5 IU/kg/d	90	1.11
		100 IU/mL	Regular (Vial)	0.5 IU/kg/d	95	
	Glargine + Aspart	100 IU/mL	Glargine (Pen)	0.5 IU/kg/d	90	1.34
		100 IU/mL	Aspart (Pen)	0.5 IU/kg/d	95	
	Glargine + Glulisine	100 IU/mL	Glargine (Pen)	0.5 IU/kg/d	90	4.79
		100 IU/mL	Glulisine (Pen)	0.5 IU/kg/d	70	

Note: The weight 75 kg was considered in calculations.

 In all the assessed therapeutic options, the treatment was affordable only in the case of NPH insulin with regular or aspart insulin. The glulisine and detemir insulin and lispro were not affordable in all the approaches since lispro was not covered by the insurance, and the detemir coverage is 70% with the affordability ratio of 3.45.

## Discussion

 This study was conducted based on the WHO/HAI methodology to determine the affordability of diabetes medication therapy for the Iranian population. The first main finding which the results revealed was that all the registered oral antidiabetic medications used in the mono, dual and triple therapies in Iran were affordable for diabetes 2 patients, in the initial treatment and even those with increased doses, because of their low price as well as 70% insurance coverage. It seems this finding is in line with aim 9 of Iranian National Service Framework for Diabetes, which the aim of 80% access to generic essential medicine and technologies in the public and private sectors was targeted for health system.^39^

 In line with our finding, a 12-year-long retrospective study in Iran, which investigated the affordability of essential diabetes medicines, demonstrated that the costs of treatment with metformin, glibenclamide, gliclazide, repaglinide, and pioglitazone or even the combination therapy were affordable for the diabetes patients (the cost of the combination therapy was about half a minimum daily wage).^22^

 The second main finding which needs more attention was on affordability of insulin therapy. Our study revealed that the intermediate-acting insulins such as NPH, regular, aspart insulins were affordable while none of the long-acting insulins were affordable. In simple word, medication therapy in patients with type 2 diabetes would be unaffordable only if the oral therapy failed and insulins such as detemir, glargine, or glulisine were added to the treatment regimen. These insulins also are non-affordable in type 1 diabetes. However, there are some limitations regarding using intermediate-acting insulins, such as NPH, including the interlay variation in absorption after injection and the peak-action profile. Basal insulin analogs have the advantage of lower risk of hypoglycemia in patients with type 1 and type 2 diabetes and also improved glycemic control.^40^ Cost-related medications non-adherence is associated with higher rate of all-cause mortality and cardiovascular complications among newly diagnosed type 2 diabetes mellitus patients.^41^ Although more adherent patients incur higher pharmacy costs, these are generally offset by savings in other areas such as costs of hospital admissions and physicians’ visits.^42^

 Thus, the patients have to use NPH, as the basal insulin component. glargine duration of action is 24 hours and no pronounced peak is seen with this type of insulin.^43^ The studies have shown that the risk of non-adherence to medications and consequently, poorly controlled diabetes is higher in patients with financial burdens related to diabetes.^44^

 This is while insulins are considered as an effective treatment for optimal glycemic control in patients with type 1 diabetes mellitus and some type 2 diabetes mellitus, whose disease cannot be treat by oral medicines^45,46^ and enjoy an acceptable insurance coverage in Iran.^21^ In line with our findings, Sarayani et al showed that, among insulins, only regular and intermediate-acting insulin were constantly affordable while the premixed insulin became affordable in the last 3 years of their study (2010-1012).^22^ Novel insulin preparations such as premixed aspart insulin and combination of aspart and glargine were constantly non-affordable during the study period (2000-2012).^22^ In 2012, the cost of the combination therapy with aspart and glargine insulin was one day more than that of the premixed aspart insulin in terms of the least daily wages (5.8 to 4.8 of the least daily wages).^22^ It is worth noting that the insulins affordability further improved due to memorandum of understanding of health ministry with health insurance organizations as the affordability ratio in the present study was in the range of 0.02-3.45 for all insulins, except for Lispro with affordability ratio of 70 as it is under no insurance coverage.

 In 2018, the Insulin Access and Affordability Working Group published their study and concluded that the prices of insulin had increased a few years before their study time, and that the average price of insulin was nearly tripled during 2002-2013.^47^ Furthermore, there was a shift in insulin utilization from the less expensive human insulins to more expensive human insulin analogs such as glargine, aspart, glulisine, detemir, and lispro during the past decade, and this affected the total costs of insulin.^47^ Nowadays, the high prices of insulins made these important antidiabetic agents non-affordable in many countries, even in high-income and developed countries.^48^ In 2015, 15 availability and price surveys were conducted in 13 LMICs.^49^ The results showed that insulins were less affordable than metformin and gliclazide.^49^ For example, regarding isophane as a human insulin in the concerned countries, only 3 countries (namely Brazil, Kyrgyzstan, and Pakistan) achieved the goal of WHO Global Action Plan for Prevention and Control of NCDs 2013-2020, ie, 80% availability of the affordable essential medicines.^49,50^ The least affordable insulins were long-acting analogues and none of the countries reached the defined target in terms of glargine.^49^ It was also observed that analogues were substantially more expensive than human insulin in all 3 sectors, namely public and private pharmacies and private hospitals/clinics.^49^

 In another study, the data from 30 surveys in 20 LMICs, including Iran, conducted from 2008 to 2015 was analyzed.^51^ According to this study in the public and private sectors of low-, lower-middle-, and upper-middle-income countries, 17%, 21%, and 45% of diabetes medicines were available (80% or greater) and affordable. The equivalent percentages for the private sector were 28%, 23%, and 32%, respectively. Regarding metformin, it was both available and affordable in the public and private sectors in Mauritius, Lebanon, Iran, Colombia, India (Delhi), and Afghanistan. Consistent with our study, this study demonstrated that the affordability of metformin in Iran was relative.^51^

 Moreover, the evidence from a Prospective Urban Rural Epidemiology (PURE) study in 22 countries indicated poor availability and affordability of essential diabetes medicines, including metformin, sulfonylureas (namely gliclazide and glibenclamide), and insulin.^32^ Further, 13.8% and 36.7% of households suffering from diabetes could not afford metformin and insulin for monthly supplies (defined as >20% of the threshold of their capacity to pay).^32^ In a subset of a PURE study, Attaei et al assessed the availability and affordability of adding metformin to high blood pressure medications in LMCI to detect the households’ monthly capacity to pay.^52^ The results revealed that the affordability decreased for the households in the combination therapy. Regarding this threshold, unaffordable households are defined as those that their total monthly expenditure for the cheapest medicines of the combination therapy goes beyond 20% of the households’ capacity to pay. In this assessment, the basic subsistence needs such as household expenditures on food are deducted from the monthly household income, and the household expenditures on housing and transportation are subtracted in the sensitivity analysis.^52^ However, as one of the limitations of our study, these factors were not considered in the present study. In addition, it is supposed that households bear the total cost of the medicines while a portion of the costs of the medicines was partly or fully subsidized by governments or other third parties (eg, health insurance); this issue was considered in our study.

 The emergence of newer antidiabetic agents has posed new challenges and difficulties due to the incrementing spending on the diabetes management.^17,53^ For example, among insulins, detemir and lispro were imported medications available in market since 2012 and 2006, respectively.

 Considering the aforementioned studies as well as the findings of the present study, oral medication therapies are affordable in Iran. Accordingly, policy-makers should adopt measures to improve the affordability of insulin products for diabetic patients, particularly for low-income patients, including the workers with least approved wage or even less. According to the statistics of 2017, 80% of 23 million Iranian workers which are active in various economic sectors get the lowest-paid. Moreover, some people are not full-time workers or in the informal sector, paid less than the minimum wage. In general, Iran health system is facing an access challenge of non-affordable treatment for more than 18.5 million individuals on minimal or less than minimal daily wages,^54^ they are not only susceptible to diabetes-related financial hardship but also are the most vulnerable ones. In this way, identifying non-affordable treatments, targeting these patients and protecting them from financial hardship would increase access to care and avoid catastrophic and impoverishment effects of treatment. In the case of diabetes treatment, since the limited number of patients are switched to this treatment schedules (glargine, detemir, glulisine, lispro), it seems covering and protecting the targeted patients, would not impose considerable budgets on the government. In this regard, a prospective analysis from first nationwide diabetes report of the National Program for Prevention and Control of Diabetes (2016) estimated the frequencies of insulin monotherapy and insulin combination therapy to be 1.5%, and 25.1%, respectively.^21^ It is worth to mention that not only further attention should be paid to pricing non-affordable insulins and adopting reimbursement policies to improve their affordability, but also, considering the rising costs of recent antidiabetic agents, targeting and future studies are strongly recommended to support their use as a routine treatment for diabetes.

###  Strength and Limitation 

 The key strength of this present study, to the best of the researchers’ knowledge, is that it is the first study evaluating the affordability of diabetes medication therapy in a comprehensive manner. The assessment includes all registered medicines for diabetic patients in guideline- and clinician opinion-based treatment schedules in mono-and combination therapy scenarios. However, our study had some limitations to be considered in interpreting the findings. In our study, affordability was calculated based on the costs of medicines and other medical costs while the incidental costs such as physician visits, travel, or time taken off work to visit a doctor had not been considered as such affordability might be underestimated. Furthermore, we did not consider the costs of other medicines taken by the patients and the medical costs of other comorbidities which could affect the affordability. Another limitation was that we did not evaluate the affordability of diabetes medications among different subgroups of the population separately by their income status. Similarly, we should also have accounted for the fact that the difference in access to diabetes medications, particularly the newer ones, in different regions of our country might have affected their affordability.

## Conclusion and Recommendations

 In this study, a holistic scenario-based approach based on the patients’ needs and conditions was introduced to assist countries with resource constraints in assessing their health system’s functions in improving access to medicines and strengthening it.

 It revealed that for Iranian patients with lowest paid wages, oral mono, dual and triple medication therapies maybe affordable, whereas insulin therapies may represent, in combination as well as increased doses, major barriers to treatment – in spite of their well health insurance coverages. Hence, policy-maker should consider targeting and more financial protection policies to improve the affordability of insulin therapies among this group of patients along with managing those price and promoting rational prescription and consumption.

## Acknowledgements

 We gratefully acknowledge the assistance provided by clinicians, who shared their clinical experience in diabetes management.

## Ethical issues

 This study received full ethical approval (IR.SUMS.REC.1396.S1051).

## Competing interests

 Authors declare that they have no competing interests.

## Authors’ contributions

 NM, MKH, and LZ participated in the conception and design of the study. Both MM and MZ had contributed to the acquired data. LZ, PP, and NM performed analysis and interpretation of data. LZ and NM were drafted the manuscript. NM, LZ had revised the manuscript critically for important intellectual content. All authors read and approved the final manuscript.

## Funding

 This work was supported by Iran Food and Drug Administration (grant number HP-96-31).

## Authors’ affiliations

^1^Health Policy Research Center, Institute of Health, Shiraz University of Medical Sciences, Shiraz, Iran. ^2^Health Management and Economics Research Center, Iran University of Medical Sciences, Tehran, Iran. ^3^Assessment and Control of Prescribing and Use of Health Products, Food and Drug Administration, Tehran, Iran. ^4^Shiraz University of Medical Sciences, Shiraz, Iran.

## Supplementary files


Supplementary file 1. Actions performed to quantify needed medicines in treatment regimes in type 1 and 2 diabetes patients.

Supplementary file 2. Scenario-based affordability assessment for type 2 diabetes.
