# Supplementary material for: Affordability of Medication Therapy in Diabetic Patients: A Scenario-Based Assessment in Iran’s Health System Context
Source: Int J Health Policy Manag. 2020 Aug 22;11(4):443–52. doi: 10.34172/ijhpm.2020.152 (PMC9309945; doi:10.34172/ijhpm.2020.152)
Supplement: Supplementary file 1 — Actions performed to quantify needed medicines in treatment regimes in type 1 and 2 diabetes patients. [file ijhpm-11-443-s001.pdf]

Supplementary file 1

The following actions were performed to quantify needed medicines in treatment regimes in type 1 and 2 diabetes patients

For monotherapy:

1. DDD of each medicine was obtained from the WHO Collaborating Centre for drug statistics methodology (http://www.whocc.no/atc_ddd_index/).
2. Considering the fact that the antidiabetic medicine dose is based on the patient's characteristics and disease conditions, the exact dose cannot be defined for a population. Therefore, moreover DDD amount, the minimum and maximum daily dose of each medicines were defined according to the American Diabetes Association (ADA) guidelines (https://doi.org/10.2337/dc19-S009) and pharmacotherapy reference books like Applied therapeutics (https://www.amazon.com/Applied-Therapeutics-Clinical-THERAPEUTICS-KODA-KIMBLE/dp/0781748453).
3. The majority of patients with diabetes require lifelong treatment due to defects in their pancreas, so all antidiabetic medicines were considered as chronic disease medicines and the calculations were performed based on a treatment period of at least 1 month.
4. The amount of out of pocket payment by patients for each medicine, was calculated regarding the amount of insurance coverage and extra financial protection such as MOU between Iran FDA and health insurance organizations (http://irc.fda.gov.ir/nfi).
5. The treatment cost was calculated according to the least price of generic medicine (http://irc.fda.gov.ir/nfi).
6. Calculations were based on the local currency (Iranian Rial).
