# Supplementary material for: Affordability of Medication Therapy in Diabetic Patients: A Scenario-Based Assessment in Iran’s Health System Context
Source: Int J Health Policy Manag. 2020 Aug 22;11(4):443–52. doi: 10.34172/ijhpm.2020.152 (PMC9309945; doi:10.34172/ijhpm.2020.152)
Supplement: Supplementary file 2 — Scenario-based affordability assessment for type 2 diabetes. [file ijhpm-11-443-s002.pdf]

Supplementary file 2. Scenario-based affordability assessment for type 2 diabetes

Therapy Phase	NO	Generic Name		Dose	Dosage Form	Insurance coverages (%)	Dose of Administration/day	Treatment response	Affordability ratio
**Mono Therapy**	1	Metformin		500 mg	TABLET	70	500mg/day	Yes	0.02
							The dose is increased by 50% every 7 days	No	0.06
	2	Glibenclamide		5 mg	TABLET	70	2.5mg/day	Yes	0.004
							The dose is increased by 50% every 7 days	No	0.01
	3	Gliclazide		80 mg	TABLET	70	40mg/day	Yes	0.01
							The dose is increased by 50% every 7 days	No	0.03
**Dual Therapy**	1	Metformin+Acarbose	Metformin	500 mg	TABLET	70	1500mg/day	Yes	0.09
			Acarbose	50 mg	TABLET	70	25mg/day		
			Metformin	500 mg	TABLET	70	1500mg/day	No	0.1
			Acarbose	50 mg	TABLET	70	50mg/day		
	2	Metformin+Repaglinide	Metformin	500 mg	TABLET	70	1500mg/day	Yes	0.38 and

			Repaglinide	1 mg	TABLET	70	base on HgA1C<br><8% 0.5 tid<br>>8% 1tid		0.22
			Metformin	500 mg	TABLET	70	1500mg/day	No	0.80
			Repaglinide	1 mg	TABLET	70	16 mg/day		
	3	Metformin+Glibenclamide	Metformin	500 mg	TABLET	70	1500mg/day	Yes	0.07
			Glibenclamide	5 mg	TABLET	70	2.5mg/day		
			Metformin	500 mg	TABLET	70	1500mg/day	No	0.07
			Glibenclamide	5 mg	TABLET	70	12.5mg/day		
	4	Metformin+Gliclazide	Metformin	500 mg	TABLET	70	1500mg/day	Yes	0.08
			Gliclazide	80 mg	TABLET	70	40mg/day		
			Metformin	500 mg	TABLET	70	1500mg/day	No	0.10
			Gliclazide	80 mg	TABLET	70	200mg/day		
	5	Metformin+Pioglitazone	Metformin	500 mg	TABLET	70	1500mg/day	Yes	0.11
			Pioglitazone	30 mg	TABLET	70	15mg/day		
			Metformin	500 mg	TABLET	70	1500mg/day	No	0.19
			Pioglitazone	30 mg	TABLET	70	75mg/day		

**Triple therapy**	1	Metformin+ Pioglitazone+ Glibenclamde	Metformin	500 mg	TABLET	70	1500mg/day	Yes	0.12
			Pioglitazone	30 mg	TABLET	70	15mg/day		
			Glibenclamide	5 mg	TABLET	70	2.5mg/day		
			Metformin	500 mg	TABLET	70	1500mg/day	No	0.20
			Pioglitazone	30 mg	TABLET	70	75mg/day		
			Glibenclamide	5 mg	TABLET	70	12.5mg/day		
	2	Metformin+ Pioglitazone+ Gliclazide	Metformin	500 mg	TABLET	70	1500mg/day	Yes	0.13
			Pioglitazone	30 mg	TABLET	70	15mg/day		
			Gliclazide	80 mg	TABLET	70	40mg/day		
			Metformin	500 mg	TABLET	70	1500mg/day	No	0.23
			Pioglitazone	30 mg	TABLET	70	75mg/day		
			Gliclazide	80 mg	TABLET	70	200mg/day		
	3	Metformin+ Pioglitazone+ Acarbose	Metformin	500 mg	TABLET	70	1500mg/day	Yes	0.14
			Pioglitazone	30 mg	TABLET	70	15mg/day		
			Acarbose	50 mg	TABLET	70	25mg/day		
			Metformin	500 mg	TABLET	70	1500mg/day	NO	0.23
			Pioglitazone	30 mg	TABLET	70	75mg/day		

			Acarbose	50 mg	TABLET	70	50mg/day		
	4	Metformin+ basal Insulin**	Metformin+ Insulin isophane	500 mg	Metformin	70	1500mg/day	Yes	0.24
				100 IU/ml	Insulin isophane	90	0.4IU/kg/day		
			Metformin+ Insulin glargine	500 mg	Metformin	70	1500mg/day	Yes	0.78
				100 IU/ml	Insulin glargine	90	0.4IU/kg/day		
			Metformin+Insulin detemir	500 mg	Metformin	70	1500mg/day	Yes	2.65
				100 IU/ml	Insulin detemir	70	0.4IU/kg/day		
**Insulin Therapy**	5	50% Basal+ 50% Rapid or Short -Acting	NPH+Regular	100 IU/ml	Insulin isophane	90	0.5IU/kg/day	Lifetime	0.34
				100 IU/ml	Regular	95	0.5IU/kg/day		
			NPH+ Aspart	100 IU/ml	Insulin isophane	90	0.5IU/kg/day	Lifetime	0.53
				100 IU/ml	Aspart	95	0.5IU/kg/day		
			NPH+ Glulisine	100 IU/ml	Insulin isophane	90	0.5IU/kg/day	Lifetime	1.89

				100 IU/ml	Glulisine	70	0.5IU/kg/day		
			Glargine+ Regular	100 IU/ml	Glargine	90	0.5IU/kg/day	Lifetime	0.88
				100 IU/ml	Regular	95	0.5IU/kg/day		
			Glargine+ Aspart	100 IU/ml	Glargine	90	0.5IU/kg/day	Lifetime	1.07
				100 IU/ml	Aspart	95	0.5IU/kg/day		
			Glargine+ Glulisine	100 IU/ml	Glargine	90	0.5IU/kg/day	Lifetime	2.43
				100 IU/ml	Glulisine	70	0.5IU/kg/day		

Note: all calculation were performed for patient of 75 kg.

** If not responded, shift to insulin therapy. See this part.
